# Health effects of loss of function variants in gene targets for Alzheimer’s disease prevention

**DOI:** 10.1007/s10072-025-08578-w

**Published:** 2025-10-22

**Authors:** Yuan Ma, Allena Zhang, Sabrina Asri, David Curtis

**Affiliations:** UCL Genetics Institute, University College London, Darwin Building, Gower Street, London, WC1E 6BT UK

**Keywords:** Alzheimer’s disease, Coding variant, APP, MAPT, APOE

## Abstract

Treatments for Alzheimer’s disease (AD) have to date met with limited success but potentially promising approaches involve attempting to reduce expression of one of the genes coding for key proteins involved in AD pathogenesis. Potential targets for such interventions include *APOE*, *APP* and *MAPT* but there is little published evidence to help assess whether reducing expression of one of these genes might cause adverse effects. We investigated 470,000 exome-sequenced UK Biobank participants to identify those carrying a naturally occurring loss of function (LOF) variant in these genes to see if they had impaired general health or educational attainment. For *APOE* we additionally tested for association with hyperlipidaemia and for *APP* and *MAPT* we tested for associations with mental health and epilepsy. For *APOE*, *APP* and *MAPT* there were respectively 56, 118 and 333 carriers of LOF variants. For each gene, there was no association between carrying a LOF variant and any of the phenotypes tested and for some phenotypes confidence intervals of effect size were small, ruling out any large effect. The findings provide some reassurance that interventions aimed at reducing expression of one of these genes may not necessarily be associated with significant adverse effects, though the small number of phenotypes examined means we cannot be confident that such treatments would be completely safe. Since LOF variants are very rare, further investigation of the effects of reduced gene expression can be undertaken within the context of clinical trials. This research has been conducted using the UK Biobank Resource.

## Introduction

Modifying the progression of Alzheimer’s disease (AD) represents an important challenge to medicine. While at time of writing two treatments have been licensed, lecanemab and donanemab, these monoclonal antibodies against Aβ residues carry significant risks of adverse events, particularly amyloid-related imaging abnormalities (ARIA) [1]. Although donanemab has exhibited efficacy in clearing Aβ plaques, in clinical trials there were three reported deaths linked to ARIA [2]. It is also argued that these treatments have failed to demonstrate substantial clinical benefits [3]. For instance, in the UK although donanemab and lecanemab have been licensed they have not been approved by the National Institute of Health and Care Excellence (NICE), primarily due to concerns about cost-effectiveness, and this means that they are not available through the National Health Service [4, 5].

Rather than using antibodies to promote the removal of proteins associated with AD, an alternative strategy could target the production of these proteins. Potentially this might be achieved by manipulating the expression of the genes coding for proteins implicated in AD pathogenesis, offering the possibility of safer and more effective treatments. The protein products of three key genes play important roles in the pathogenesis of AD: *APP*, *MAPT* and *APOE*. Rare nonsynonymous coding variants in *APP* can cause presenile AD and *APP* codes for amyloid-beta precursor protein, which undergoes cleavage to form Aβ fragments and these aggregate to form the amyloid plaques which are a hallmark of AD [6]. The other characteristic histological feature of AD is the observation of neurofibrillary tangles, consisting largely of hyperphosphorylated tau protein and this is coded for by *MAPT* [7–9]. Abnormalities of tau protein processing feature in a number of neurodegenerative diseases, collectively termed tauopathies, and some of these are caused by variants in *MAPT* [10]. Three allelic forms of *APOE*, termed *APOE2*, *APOE3* and *APOE4*, have major effects on risk of AD, with the *APOE4* increasing risk relative to the commoner *APOE3* allele and with *APOE2* being somewhat protective [11, 12]. The best evidence to date suggests that the increased risk associated with *APOE4* results from a combination of both loss of function and toxic gain of function effects, implying that reducing levels of *APOE4* expression might be protective against AD [13]. Thus *APP*, *MAPT* and *APOE* all represent potential targets for genetically based therapies against AD.

Some therapeutic interventions targeting these genes are already in development. In mice carrying a copy of the human *APP* gene with mutations causing higher expression, a small interfering RNA (siRNA) targeting *APP* was shown to reduce *APP* mRNA and protein levels, as well as subsequent Aβ deposition [14]. This led to the development of ALN-APP, a siRNA which targets APP, and in an early clinical trial in humans this has been shown to result in lower levels of soluble APPβ, Aβ40 and Aβ42 [15]. In a study of mice carrying the pathogenic *Mapt* P301S mutation, an antisense oligonucleotide (ASO) directed against *Mapt*, referred to as Tau^ASO−12^, was able to lower tau mRNA and protein levels and resulted in a reduction in subsequent tau aggregation [16]. Following on from this, BIIB080, a *MAPT*-targeting ASO, has been developed for humans and early trials show that it seems to be well tolerated and results in reduced tau accumulation relative to placebo [17, 18]. Clinical trials of BIIB080 are ongoing, while early phase trials are being carried out for another *MAPT*-targeting ASO, NIO752 (https://clinicaltrials.gov/study/NCT05469360 and https://clinicaltrials.gov/study/NCT06372821). *APOE4*-targeting ASOs reduced tau pathology in mice carrying *Mapt* P301S but currently there are no trials of *APOE*-targeting therapies in humans [13, 19].

While gene-based therapies targeting *APP*, *MAPT* and *APOE* seem worth exploring further, an obvious consideration is that reducing expression of these genes might have adverse effects. This issue can potentially be explored in animal studies but another approach is to study people who have naturally occurring DNA sequence variants causing loss of function (LOF) of these genes.

Homozygous APP-deficient mice are viable and fertile but have somewhat reduced locomotor activity and are prone to reactive gliosis [20]. They also fail to increase cerebral blood flow in response to hypoxia and have markedly increased mortality as a result of experimentally induced cerebral ischemia [21]. There is a single case report of a 20-month-old male infant homozygous for an *APP* LOF variant who exhibited significant developmental delay, microcephaly, and severe epilepsy [22]. Both heterozygous parents were phenotypically normal. However a maternal uncle was reported to have generalised seizures and the paternal grandfather had died aged 40 with hydrocephalus. Neither of these relatives was genotyped but each would have a 50% chance of carrying the variant. The same study reported that the Exome Aggregation Consortium (ExAC) database contained seven additional heterozygous carriers of *APP* LOF variants but no phenotypic information was available for them.

Early studies of tau knockout (KO) mice noted no overt phenotype when young but some muscle weakness and impaired motor function in older mice [23]. Acute knockdown of tau in the adult hippocampus significantly impairs motor coordination and spatial memory [24]. Tau deletion can also lead to anxiety-related behavior and impaired contextual and cued fear memory [25]. Tau reduction decreases hyperexcitability in both mouse and Drosophila genetic models of epilepsy [26]. There are no reports of humans carrying LOF variants in *MAPT*.

Animal studies have demonstrated that homozygous *ApoE*-deficient mice have neurodegenerative changes, synaptic deficits, and cognitive impairments and that these abnormalities also are seen if *ApoE* is selectively knocked out only in the brain [27, 28]. Brain alterations due to homozygous knockout of *ApoE* are observable from birth and include synaptic deficits, neuroinflammation, and changes to levels of APP and tau [29]. As well as neurodevelopmental abnormalities, *ApoE* deficient mice also have metabolic abnormalities including severe hypercholesterolemia and atherosclerosis [30, 31]. Four members of the same family with homozygous familial APOE deficiency had severe dyslipidemia, type III hyperlipoproteinemia, and premature cardiovascular disease [32]. However, heterozygous carriers (*n* = 10) maintained nearly normal plasma lipid levels, with mean plasma APOE protein concentrations reduced to approximately 42% of normal, suggesting that a single functional *APOE* allele may be sufficient for lipid homeostasis. A study of 56,864 individuals from the Alzheimer’s Disease Sequencing Project (ADSP) was able to identify seven individuals who were heterozygous carriers of *APOE* LOF variants [33]. They were aged between 71 and 90 and none had major systemic health issues. Five were cognitively normal, one had progressive supranuclear palsy and one had AD. Of note, two *APOE3*/*APOE4* carriers with LOF mutations of their *APOE4* allele exhibited completely normal cognitive function at ages 79 and 90. The first had lumbar puncture at 76 years with normal levels of Aβ and tau, while the other was cognitively intact at 90 years old and, upon autopsy, showed no neuritic plaques or amyloid pathology. Given that some degree of AD pathology would be common in people carrying an *APOE4* allele, these observations suggest that reduced expression of *APOE4* may be neuroprotective.

The present study sought to investigate phenotypic effects of LOF variants in *APP*, *MAPT* and *APOE* in UK Biobank participants, who consist of approximately 500,000 volunteers who have been extensively characterised on a wide range of clinical characteristics and most of whom have undergone whole exome and whole genome sequencing [34]. This sample had previously been used to investigate rare coding variant effects on a variety of phenotypes including hyperlipidaemia [35].

## Materials and methods

The UK Biobank Research Analysis Platform was used to access the Final Release Population level exome variants in PLINK format for 469,818 exomes which had been produced at the Regeneron Genetics Center based on DNA extracted from stored blood samples and using the protocols described here: https://dnanexus.gitbook.io/uk-biobank-rap/science-corner/whole-exome-sequencing-oqfe-protocol/protocol-for-processing-ukb-whole-exome-sequencing-data-sets [34]. UK Biobank had obtained ethics approval from the North West Multi-centre Research Ethics Committee which covers the UK (approval number: 11/NW/0382) and had obtained informed consent from all participants. The UK Biobank approved an application for use of the data (ID 51119) and ethics approval for the analyses was obtained from the UCL Research Ethics Committee (11527/003).

To obtain population principal components reflecting ancestry, version 2.0 of *plink* (https://www.cog-genomics.org/plink/2.0/) was run with the options *--maf 0.1 --pca 20 approx* [36, 37].

Attention was restricted to variants with minor allele frequency (MAF) < 0.01. All exonic variants were annotated using Variant Effect Predictor (VEP) [38]. For the present analyses, the variants expected to cause loss of function (LOF) of the gene were used, consisting of stop gained, frameshift and splice site variants. The SCOREASSOC software which was used to carry out weighted burden gene-wise association analyses on this data set had assigned scores based on the LOF variants carried by each participant in the genes of interest. This score used a parabolic function of allele frequency as previously described, with extremely rare variants (MAF ~ 0) being assigned a score of 10 and less rare variants (MAF = 0.01) being assigned a score of 1 [39, 40]. LOF scores for these *APP*, *MAPT* and *APOE* had been downloaded from the UK Biobank Research Analysis Platform. For purposes of the present analyses, these scores were simply dichotomised to 1 or 0 to indicate whether a participant did or did not carry a LOF variant in each gene. The LOF variants were all rare and there no homozygotes so this process identified participants who were heterogygous carriers of LOF variants.

Because it was expected that the number of LOF variant carriers would be small, it was decided to restrict attention to only a limited number of phenotypes in order to avoid problems with multiple testing. Phenotypes were selected on the basis of their potential relevance and also using the criterion that they should be available for almost all participants. For all three genes, self-reported general health status and age finishing full time education were utilised. At recruitment, participants respond to the question: “In general, how would you rate your overall health?” Responses are recorded on a 4-point scale (1 = Excellent, 2 = Good, 3 = Fair, 4 = Poor). Participants also report the age at which they completed full time education and it was reasoned that if reduced expression of a gene resulted in some degree of neurodevelopmental impairment then this might be reflected in an average reduction in educational attainment. This measure had the advantage that it was available for nearly all participants and also, unlike any measure of current cognitive function, that it would not be affected by any later life factors which might affect cognition.

For *APOE*, because of the previously reported metabolic effects, an additional phenotype of hyperlipidaemia was included. This was defined in the same way as in the previous study which had characterised rare variant effects on hyperlipidaemia risk and incorporated information from self-report, diagnoses recorded in medical records and prescribed medication [35].

For *APP* and *MAPT* two additional phenotypes were studied, designed to investigate whether reduced expression might impact mental health or epilepsy risk. To detect possible effects on mental health, the phenotype used was referral to a psychiatrist. This phenotype was determined according to how participants had responded in their initial assessment to the touchscreen question: “Have you ever seen a psychiatrist for nerves, anxiety, tension or depression?” As discussed previously, because most mental health problems in the UK are dealt with in primary care, responding “Yes” to this question might be taken to indicate significant psychiatric morbidity [41]. Additionally, this phenotype had been shown to be genetically correlated with the diagnosis of major depressive disorder used in large genome-wide association studies of depression [41]. The epilepsy phenotype was defined in a way analogous to that used for hyperlipidaemia and based on a combination of either self-reported epilepsy or having an ICD10 code for an epilepsy diagnosis.

To test for association between LOF variants and these phenotypes, multiple regression analysis was performed using the phenotype as the dependent variable and with sex and the first 20 principal components as covariates. A likelihood ratio test was performed. The log likelihood was obtained for the null model in which the phenotype was predicted just from these covariates and this was compared with the log likelihood for the alternative model additionally including LOF carrier status, with twice the difference between log likelihoods taken to be distributed as a chi-squared statistic with one degree of freedom. Multiple linear regression was used for the quantitative phenotypes of overall health and age completed full time education while multiple logistic regression was used for the dichotomous outcomes of having hyperlipidaemia, seeing a psychiatrist or having epilepsy.

## Results

There were 56 participants who carried LOF variants in *APOE* and Table 1 shows the uncorrected summary statistics in carriers and non-carriers for the three phenotypes studied: general health, age completed full time education and hyperlipidaemia. It can be seen that the means for general health and age completed education are very similar in carriers and non-carriers, as is the percentage of participants with hyperlipidaemia. The estimates for effect sizes obtained from the multivariate analyses incorporating sex and principal components are close to null for all phenotypes, with fairly narrow confidence intervals, and for each phenotype the association with carrying a LOF variant is not statistically significant.

**Table 1:** The table shows the uncorrected summary statistics for 56 carriers of LOF variants in *APOE* compared with non-carriers along with estimates of effect size obtained from multivariate analyses incorporating sex and population principal components as covariates

Phenotype	Mean (SD) in LOF carriers	Mean (SD) in LOF non-carriers	Beta (95% CI)	*P* value
Self-reported health	2.22 (0.79)	2.14 (0.73)	0.00 (−0.02–0.02)	0.63
Age completed full-time education	17.22 (3.37)	16.72 (2.33)	0.01 (−0.07–0.09)	0.78
	**% cases in LOF carriers**	**% cases in LOF non-carriers**	**OR** **(95% CI)**	**P value**
Hyperlipidaemia	19.6%	22.6%	0.99 (0.93–1.05)	0.74

There were 118 carriers of LOF variants in *APP* and Table 2 shows the summary statistics and estimated effect sizes for the four phenotypes studied: general health, age completed full time education, referred to psychiatrist and epilepsy. Once again, it can be seen that the results for carriers and non-carriers are very similar, that estimated effects are small and that no results are statistically significant. However it should be pointed out that confidence limits for the effect size on epilepsy risk are large, a consequence of the fact that only a small percentage of participants are cases.

**Table 2:** The table shows the uncorrected summary statistics for 118 carriers of LOF variants in *APP* compared with non-carriers along with estimates of effect size obtained from multivariate analyses incorporating sex and population principal components as covariates

Phenotype	Mean (SD) in LOF carriers	Mean (SD) in LOF non-carriers	Beta (95% CI)	*P* value
Self-reported health	2.21 (0.71)	2.12 (0.77)	0.09 (−0.05–0.24)	0.19
Age completed full-time education	15.72 (4.37)	16.36 (3.44)	−0.59 (−1.33–0.14)	0.11
	**% cases in LOF carriers**	**% cases in LOF non-carriers**	**OR** **(95% CI)**	**P value**
Referred to psychiatrist	12.71%	11.44%	1.15 (0.66–1.99)	0.63
Epilepsy	1.69%	1.29%	1.34 (0.32–5.59)	0.69

There were 333 carriers of LOF variants in *MAPT* and results are shown in Table 3. Exactly as for *APP*, results are similar for carriers and non-carriers, estimated effect sizes are small and there are no statistically significant associations.

**Table 3:** The table shows the uncorrected summary statistics for 333 carriers of LOF variants in *MAPT* compared with non-carriers along with estimates of effect size obtained from multivariate analyses incorporating sex and population principal components as covariates

Phenotype	Mean (SD) in LOF carriers	Mean (SD) in LOF non-carriers	Beta (95% CI)	*P* value
Self-reported health	2.11 (0.85)	2.12 (0.77)	−0.02 (−0.11–0.06)	0.59
Age completed full-time education	16.48 (4.42)	16.36 (3.44)	0.25 (−0.23–0.72)	0.30
	**% cases in LOF carriers**	**% cases in LOF non-carriers**	**OR** **(95% CI)**	**P value**
Referred to psychiatrist	10.51%	11.44%	0.94 (0.66–1.34)	0.72
Epilepsy	1.80%	1.29%	1.46 (0.64–3.33)	0.39

## Discussion

Our results provide some reassurance that there are no obvious major health detriments associated with carrying a LOF variant in either *APOE*, *APP* or *MAPT*. Although the numbers of carriers are modest, these represent the largest samples reported to date and for some phenotypes the confidence intervals for the estimated effect sizes are small, meaning that it is unlikely that there are large effects. However we should note that because few participants had epilepsy the confidence intervals for this phenotype are large and, although we do not detect any effect, we cannot rule out the possibility that LOF variants in *APP* or *MAPT* might have a substantial effect on risk.

An obvious limitation of this study is that we have chosen to study only a very small number of phenotypes in order to avoid problems with multiple testing and of course we cannot know that there are not specific phenotypes which might be associated with reduced expression of one of these genes. While we might hope that the measure of general health could capture such an effect, we cannot rule out that some relatively rare syndrome might have increased frequency among carriers.

On the other hand, it is perhaps worth pointing out that LOF carriers are expected to have reduced gene expression on a lifelong basis whereas in the context of treatments aimed at preventing AD one might anticipate exposure only during adulthood. In the context of clinical trials, the degree to which gene expression was reduced might be more carefully titrated against beneficial and detrimental effects as these become better characterised.

Although we have been able to study only a relatively small number of LOF carriers, this did involve investigating a large sample of nearly half a million exome-sequenced participants and the small number of LOF carriers identified reflects their rarity. Thus, it does not seem likely that investigation of additional population based cohorts will throw further light on the consequences of reduced expression of these genes. Rather, it will be important to intensively follow up participants in clinical trials of interventions to reduce gene expression in order to discover any potential adverse consequences.

To conclude, our results demonstrate that carrying a LOF variant in *APOE*, *APP* or *MAPT* is not shown to be associated with a large impact on general health or on educational attainment.

## Data Availability

The raw data is available on application to UK Biobank at https://ams.ukbiobank.ac.uk/ams/. Derived variables will be returned to UK Biobank, from which they will become available in due course. Software and scripts used to perform the analyses are available at https://github.com/davenomiddlenamecurtis/UKBBscripts.20220912.
